# Supplementary figures and images for: The unique tropism of Mycobacterium leprae to the nasal epithelial cells can be explained by the mammalian cell entry protein 1A
Source: PLoS Negl Trop Dis. 2019 Mar 5;13(3):e0006704. doi: 10.1371/journal.pntd.0006704 (PMC6420055; doi:10.1371/journal.pntd.0006704)

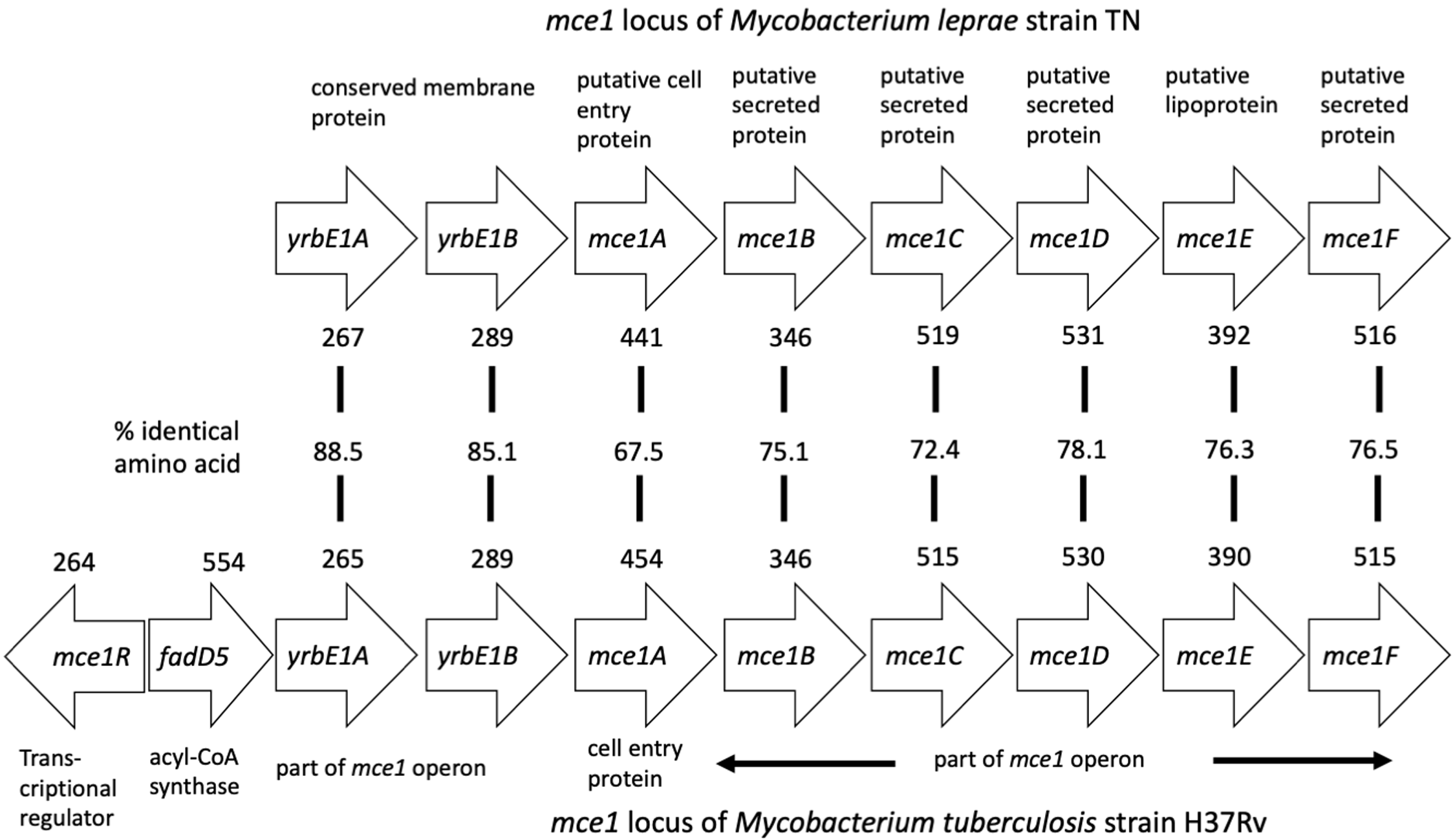

Supplement: S1 Fig — The genome of M. tuberculosis strain H37Rv contains four mce operons (mce1, 2, 3 and 4), however, only one mce operon is conserved in the genome of M. leprae strain TN. The mce1 operon is comprised of 8 gene (yrbE1A, yrbE1B, mce1A, mce1B, mce1C, mce1D, mce1E and mce1F). M. leprae mce1A gene is highly homologous (67.5%) to M. tuberculosis mce1A gene associated with mammalian epithelial cell entry and intracellular survival inside macrophages. Upper and bottom columns indicate the genes in mce1 locus of M. leprae and M. tuberculosis, respectively. The numbers representing in upper and bottom columns indicate amino acid residues corresponding to each gene. The numbers representing in middle column indicate percentages of amino acid identical to each set of genes. All of genetic information indicated in this figure were obtained from the web sites of Institut Pasteur (http://genolist.pasteur.fr/) and Sanger Centre (http://www.sanger.ac.uk/). (TIF) [file pntd.0006704.s001.tif]

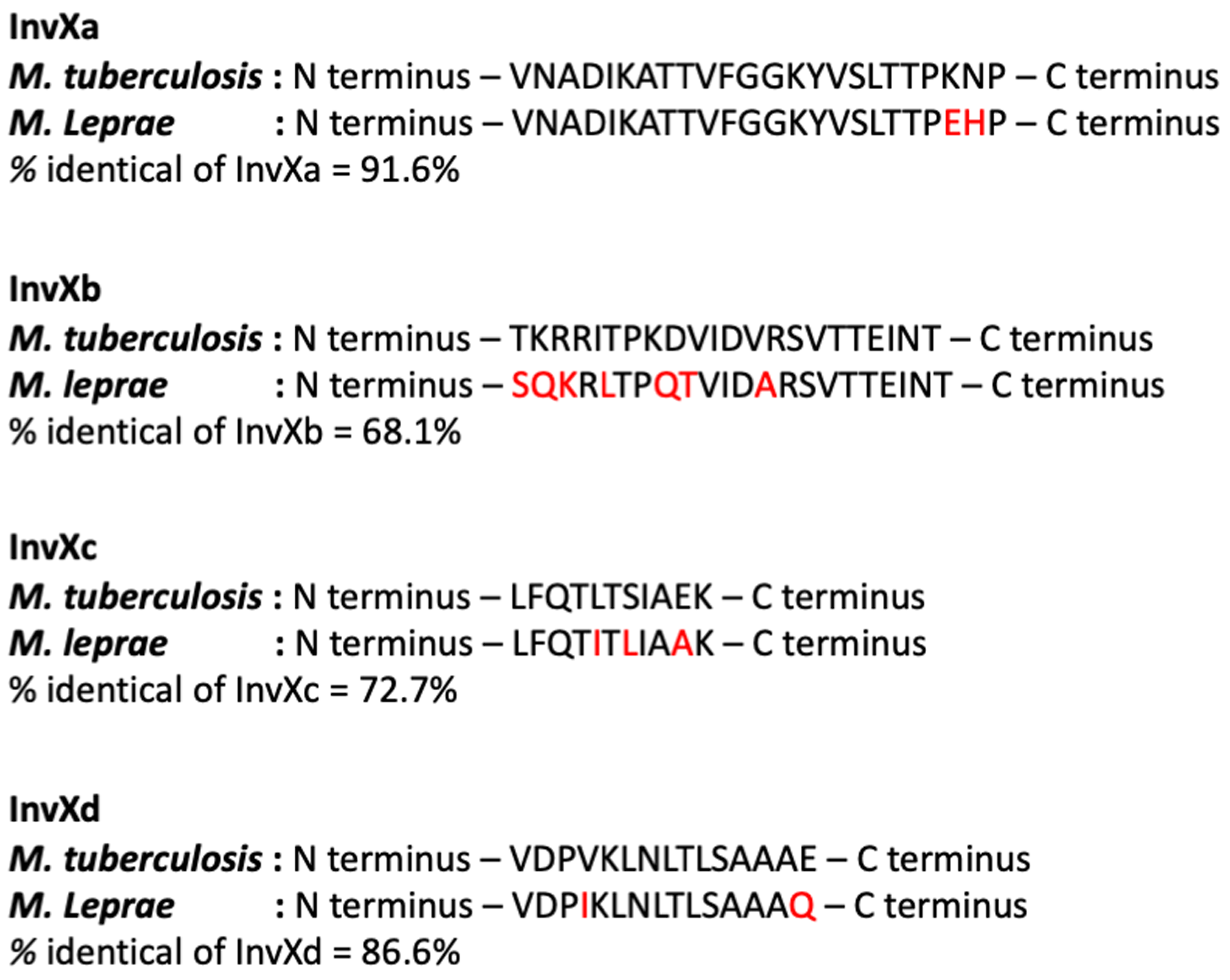

Supplement: S2 Fig — (TIF) [file pntd.0006704.s002.tif]

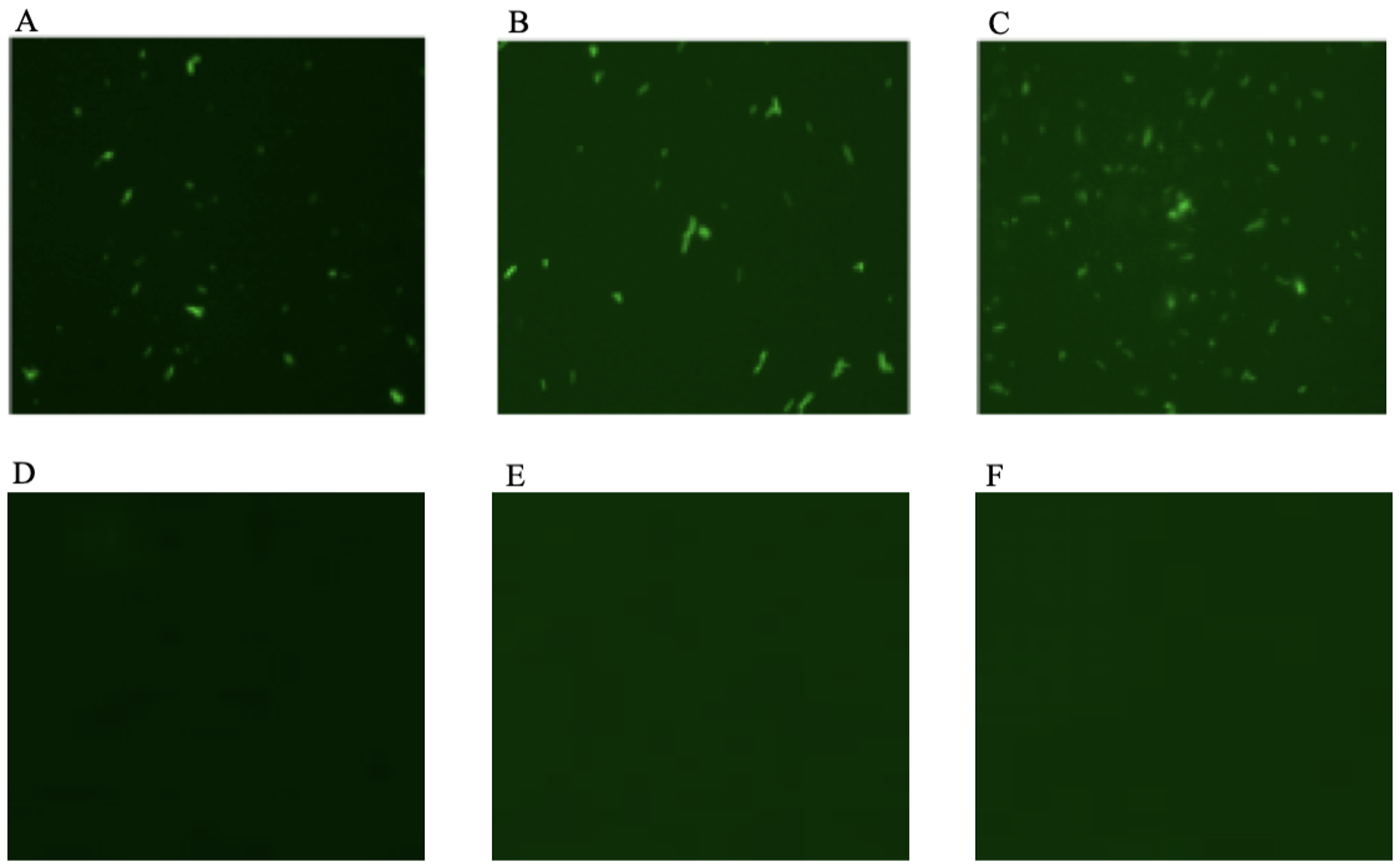

Supplement: S3 Fig — invX, invY and invZ is presented on the surface of E. coli cells by the AIDA autotransporter translocator. E. coli cells were surface labeled with a mouse polyclonal Ab raised against Mce1A and a FITC-conjugated anti-mouse secondary Ab. Fluorescence microscopy showed that E. coli expressing the InvX, InvY and InvZ -AIDA fusion protein bound anti-Mce1A Ab. (A) InvX, (B) InvY, (C) InvZ. (D), (E) and (F) are controls of InvX, InvY and InvZ, respectively where normal mouse serum is used. (TIF) [file pntd.0006704.s003.tif]

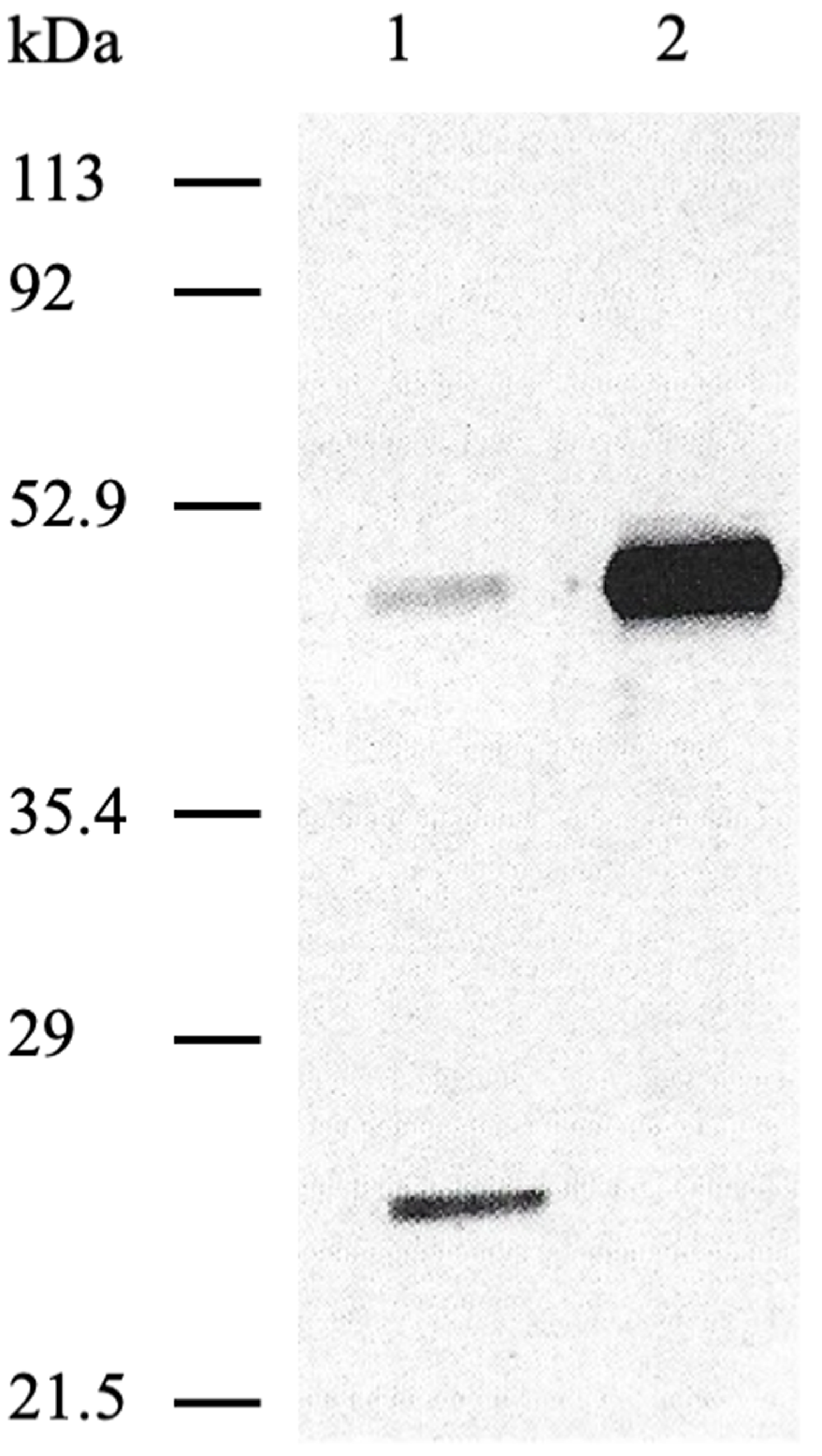

Supplement: S4 Fig — The mouse hyperimmune serum raised against r-lep45 kDa recognizes 27 and 45 kDa proteins in the whole cell lysates of M. leprae strain Thai 53 (lane 1). The r-lep45 kDa which the serum was raised against was used as a positive control against and is recognized as shown in lane 2. (TIF) [file pntd.0006704.s004.tif]
